# The impact of child abuse: neuroscience perspective

**DOI:** 10.3325/cmj.2015.56.315

**Published:** 2015-06

**Authors:** Lukas M. Konopka

**Affiliations:** Department of Psychiatry, Loyola Medical Center, Maywood Il, and Chicago Brain Institute, Rolling Meadows, Il, USA

The world acknowledges the vulnerability of children and the crucial necessity for their oversight, nurturing, and love, but how we form the hearts and minds of children remains a worldwide challenge. A child’s mind begins with their brain, and their brain begins with a structure morphologically similar to an adult, yet to reach the functional integrity of an adult, a child’s brain must undergo approximately thirty years of evolution (1). Neurodevelopmental research has taught us about specific windows of neural development that exist for the optimal wiring of children’s sensory systems. During these windows, a child’s sensory systems learn to appropriately process perceptions and, ultimately, integrate them into fully developed human cognition (2). During brain development, how children play their environmental instruments and respond to their life’s music will depend on the score of their individual genetic vulnerabilities. Eventually, this mental evolution begins to shape who they are, how they view the world, how they interact with others, and their attitudes toward self. Therefore, developmental disruptions can cause significant consequences and the literature clearly shows that early neglect, nutrition, socialization, and stress define who we are as children and become as adults. Children’s significant dependence upon caregivers clearly can leave them at risk for deficient cognitive development. In addition, their very active limbic systems teach them about what is or is not safe, so with early threats and adversity, they begin to develop survival strategies that may endure for a lifetime. Child abuse, whether physical, psychological, or sexual, presents a threat that is universally recognized as having a significant negative impact on the life of a child (3). Nevertheless, overwhelming data exist suggesting that child abuse, particularly sexual abuse, currently occurs throughout the world. The ramifications of this fact are enormous (4).

Many neuroscientific studies of structural and functional brain networks that compare abused and non-abused children demonstrate the significant results of childhood sexual abuse. These studies have uncovered electrophysiological brain changes such as abnormal neurophysiological interactions (5,6), altered brain structure, and deregulated brain activation to stimuli (7). In addition to the anatomical and electrophysiological consequences, abused children show significant cognitive impairments that can impact their future life trajectories as they emerge into adulthood (8). Psychiatric disorders also are more common in abused children (9). Often children who have been sexually abused develop behaviors that lead to disruptive relationships within their families. They may also engage in self-harm or other aberrant behaviors and sometimes become perpetrators of abuse as a result of their early experiences of helplessness and fear (10,11). When these adults gain power and authority, they can extend their aggression to unsuspecting innocent victims. Other data suggest that the individuals most vulnerable to abuse are those who are mentally or physically compromised (12). Sexual abuse often occurs when the vulnerable have no resources, particularly when they are entrusted to unsuspected abusers. Such cases have come to light in the recent reports of clerical sexual abuse where clergy use their spiritual authority to gain a person’s trust and confidence (13). Clergy naturally command authority and foster the trust that gives a potential abuser free access to power over another. Stories of clerical abuse and its tremendously damaging impact are surfacing throughout the world. For the abused, the abuse often lingers for many years, silently hijacking the choices and trajectories of their lives: in many cases, abuse dominates the lives of its victims because they feel helpless and shamed into silence (14). Often, victims will deny their trauma until investigations expose the abuse which, unfortunately, forces the victim to relive their painful memories and trauma.

Clearly, childhood sexual abuse significantly changes its victim’s brain and alters its function, cognition, and emotion. Admittedly, abuse has differing effects that depend on an individual’s genetic vulnerabilities and familial support. Nevertheless, the abused often confront the additional challenges of shame and society’s belief that partial guilt rests with the victim. Therefore, to help victims heal, we must vigorously challenge the humiliation that still exists in the public narrative. They deserve the chance to strive for independence and a trauma-free future by regaining their integrity through familial and societal reconciliation.
